# Acupuncture combined with auricular acupressure for dry eye: a SPIRIT-guided protocol for a multicenter randomized controlled trial

**DOI:** 10.3389/fmed.2026.1738110

**Published:** 2026-02-12

**Authors:** Zhuoyi Li, Yika Mou, Shiyun Sun, Luyao Fu, Lingqian Chen, Qin Guo, Jing Liu, Ruijie Ma

**Affiliations:** 1 The Third School of Clinical Medicine (School of Rehabilitation Medicine), Zhejiang Chinese Medical University, Hangzhou, Zhejiang, China; 2 Department of Acupuncture and Moxibustion, Third Affiliated Hospital of Zhejiang Chinese Medical University, Hangzhou, Zhejiang, China

**Keywords:** acupuncture, auricular acupressure, dry eye disease, multicenter, randomized clinical trial, study protocol

## Abstract

**Introduction:**

Dry eye disease (DED) is a chronic disorder of the ocular surface (OS) that is influenced by a variety of factors, resulting in ocular discomfort and visual impairment. Studies have also shown that dry eye syndrome detrimentally impacts patients’ sleep quality (SQ), mental well-being, and overall mental health. This SPIRIT- and STRICTA-compliant protocol describes a multicenter, single-blind randomized controlled trial designed to evaluate the efficacy and safety of acupuncture combined with auricular acupressure compared with 0.1% sodium hyaluronate eye drops in adults with DED.

**Methods and analysis:**

A total of 126 patients with DED will be recruited and randomly assigned to one of two groups at a 1:1 ratio across three centers. Participants in the intervention group will receive acupuncture combined with auricular acupressure for 8 weeks, whereas the control group will be treated with 0.1% sodium hyaluronate eye drop. Following the intervention, an eight-week follow-up will be conducted. Primary outcome is the Ocular surface disease Index (OSDI). Secondary outcomes are Breaking up time (BUT), Conjunctival Impression Cytology (CIC), cornea fluorescein staining (CFS), Tear meniscus height (TMH), Meibum quality score (MQS), Meibomian gland score (MGS), Meiboscore, Function of the meibomian gland, Pittsburgh Sleep Quality Index (PSQI), Self-rating depression Scale (SDS), and Self-rating anxiety scale (SAS). The primary endpoint is the change in OSDI score from baseline to week 8, and the primary effect measure will be the adjusted between-group mean difference, estimated using a linear mixed-effects model. This study will assess whether acupuncture combined with auricular acupressure improves symptoms of dry eye and affects sleep and mood compared with sodium hyaluronate.

**Clinical trial registration:**

https://itmctr.ccebtcm.org.cn, Identifier ITMCTR2024000673.

## 1 Introduction

DED is a widespread ocular condition necessitating prompt diagnosis and thorough therapeutic management. The 2020 Chinese Expert Consensus on Dry Eye (1) describes DED as a chronic OS disorder stemming from multiple etiological factors. This ailment may trigger a variety of ocular discomforts and visual deficits due to tear film instability or disruptions in the OS microenvironment. Common manifestations of DED include redness, eye fatigue, tingling sensations, dryness, a feeling of a foreign body, and compromised vision (2). According to the TFOS Dry Eye Workshop II and the updated TFOS DEWS III, the global prevalence of DED ranges widely and continues to increase, particularly among aging populations and women (3). Notably, the prevalence of DED escalates with age and predominantly affects women more than men, with an onset age that is decreasing (4, 5). DED adversely impacts the quality of life (QoL) and productivity, increasing both familial economic strains and the demands on healthcare systems (6).

Tear film instability, hyperosmolarity, inflammation, and cellular damage are acknowledged as the primary pathological mechanisms driving the adverse cycle associated with DED (1). The aim of DED treatment is to restore OS and tear film homeostasis by disrupting this cycle (7). Conventional therapies typically encompass a variety of artificial tears, eyelid margin hygiene practices, meibomian gland massages, intense pulsed light therapy, thermotherapy, or thermal pulsation, and may extend to topical or systemic anti-inflammatory treatments and surgical procedures (8, 9). Nevertheless, these methods are frequently criticized for their prohibitive costs, limited efficacy in symptom alleviation, and transient beneficial effects.

Acupuncture is widely recognized as a form of complementary and alternative medicine that involves inserting fine needles at specific points known as acupuncture points. Numerous clinical trials have shown that acupuncture is effective for various ophthalmic disorders, including acute hordeolum, diabetic retinopathy, glaucoma, and myopia (10–12). It is also a well-established treatment for DED (13), typically targeting key acupoints such as BL2, EX-HN5, BL1, SJ23, SP6, GB20, ST2, and ST36. Studies comparing systemic versus local acupoint selection (14) have shown that whole-body acupoint therapy is more effective.

Evidence supporting acupuncture for DED originates from multiple levels, including animal mechanistic studies, small to medium-sized randomized controlled trials, and systematic reviews. Animal studies (15, 16) suggest that acupuncture may modulate lacrimal gland secretion, inflammatory cytokines, and neurosensory function; however, these findings cannot be directly extrapolated to clinical efficacy. Several RCTs and meta-analyses (17, 18) report improvements in tear film stability and patient-reported symptoms compared with artificial tears, yet heterogeneity in study design, sample size, and outcome measures remains substantial, underscoring the need for rigorously designed multicenter trials.

Auricular acupressure, a technique related to acupuncture within Traditional Chinese Medicine (TCM), involves stimulating auricular points by adhering dried seeds of vaccaria segetalis to achieve the desired effect (19). The French physician Paul Nogier, MD (1908–1996 AD), invented and promoted auriculotherapy, and pioneered the auricular mapping of the embryo’s reflection. Based on this work, Chinese acupuncturists developed ear acupuncture (20). Auricular acupressure has been proven effective in analgesia, anti-vomiting, emotion regulation (21–23), and other aspects, including DED. It targets specific auricular points such as Mu1 (TG2b) and Mu2 (AT1b) to treat DED by elevating hormones levels and regulating the sympathetic nervous system (24). This approach has proven effective for DED, both as a standalone treatment and in combination with artificial tears or electrical stimulation (25).

In addition, the impact of DED on QoL and associated mood disorders such as depression and anxiety is increasingly recognized. Chronic discomfort and pain from DED can negatively influence patients’ sleep, mood, and overall mental health (26), potentially exacerbating the severity of DED (27). In this trial, PSQI, SDS, and SAS are prespecified secondary outcomes, and analyses of these measures will be exploratory, as the study is powered for ocular symptom improvement assessed by OSDI. Researchers have found the beneficial effects of acupuncture and auricular acupressure in improving QoL and mental health (28). Bitar et al. (29) identified a strong link between DED and symptoms of anxiety and depression, proposing that effective treatments could also ameliorate these psychological issues. Therefore, we hypothesize that a combination of acupuncture and auricular acupressure might not only alleviate ocular surface symptoms but also effectively manage sleep and emotional issues in DED patients.

This multicenter, large-sample clinical, RCT is designed to assess the efficacy and safety of acupuncture combined with auricular acupressure in mitigating ocular symptoms and modulating mood and sleep among DED patients, while also ensuring the treatment’s safety and reliability through rigorous safety analyses and monitoring of adverse events.

## 2 Methods and analysis

### 2.1 Study design

**Figure 1:**
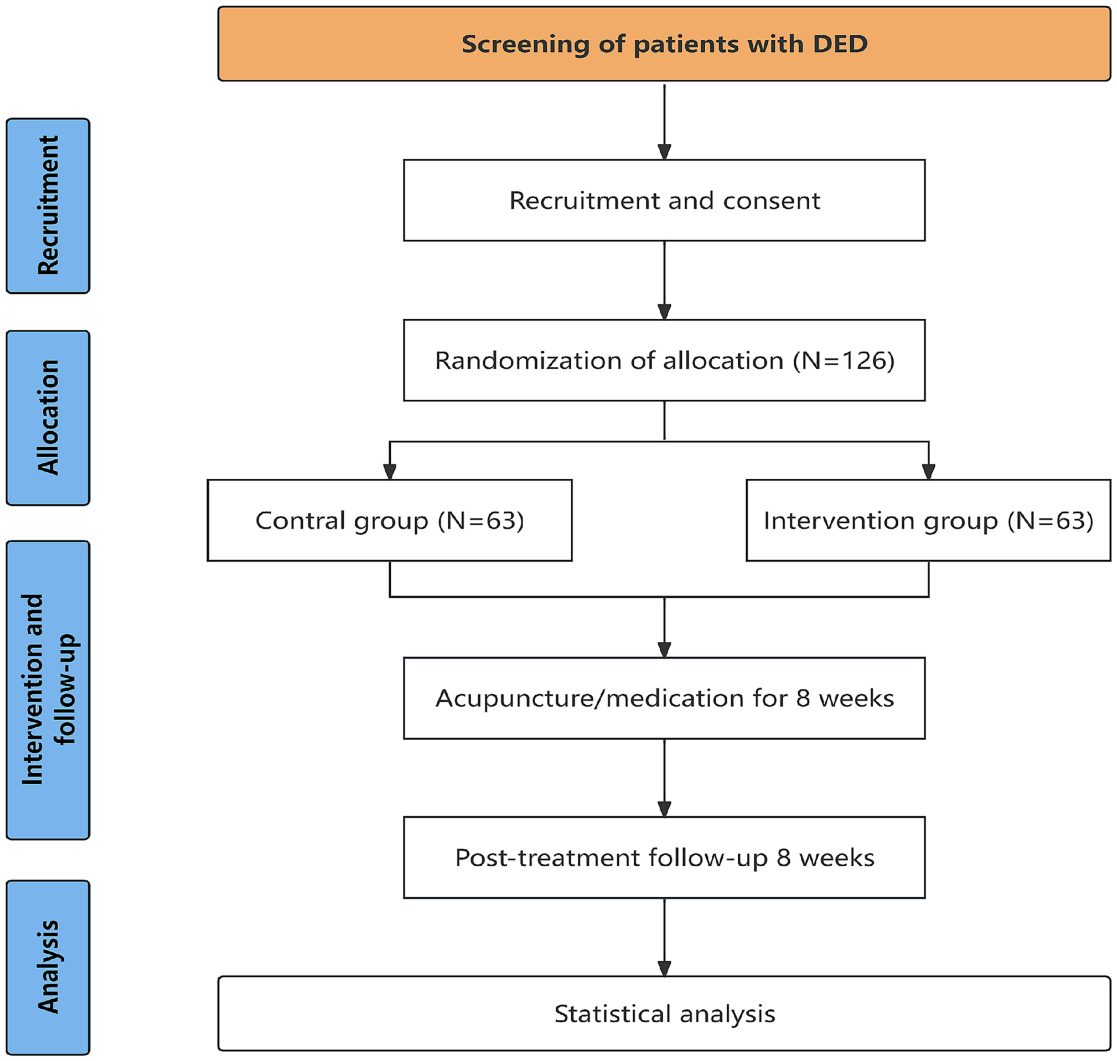
Flowchart of the research procedures.

This study is a multicenter, single-blinded, randomized controlled trial (RCT). Participants will be enlisted from three academic institutions: Third Affiliated Hospital of Zhejiang Chinese Medical University, Jiaxing Traditional Chinese Medicine Hospital, and Affiliated Optometry Hospital of Wenzhou Medical University. 126 patients will be enrolled and randomly assigned in equal proportions to the intervention or control group. Participants in the intervention group will undergo three 30-min sessions weekly over 8 weeks, whereas the control group will be treated with 0.1% sodium hyaluronate eye drop. Following the intervention, an eight-week follow-up will be conducted. Primary outcome is the Ocular surface disease Index (OSDI). Secondary outcomes are Breaking up time (BUT), Conjunctival Impression Cytology (CIC), cornea fluorescein staining (CFS), Tear meniscus height (TMH), Meibum quality score (MQS), Meibomian gland score (MGS), Meiboscore, Function of the meibomian gland, Pittsburgh Sleep Quality Index (PSQI), Self-rating depression Scale (SDS), and Self-rating anxiety scale (SAS). Evaluations will take place at weeks 0, 4, 8, 12, and 16. The enrollment schedule is illustrated in Table 1, and the flow chart of this trial is displayed in Figure 1. The completed trial will be reported in accordance with CONSORT 2010, including the extensions for non-pharmacologic treatments and harms reporting.

**Table 1:** The chart of enrollment and assessment.

Time point	Study period				
	Baseline	Treatment phase		Follow-up phase	
Enrollment	0 week	4th week	8th week	12th week	16th week
Eligibility screen	×				
Informed consent	×				
Medical history	×				
Randomization	×				
Intervention					
Experimental group		×	×		
Control group		×	×		
Assessment					
OSDI	×	×	×	×	×
BUT	×		×	×	
CIC	×		×	×	
CFS	×		×	×	
PSQI	×	×	×	×	×
SAS	×	×	×	×	×
SDS	×	×	×	×	×
TMH	×		×	×	
MQS	×		×	×	
MGES	×		×	×	
Meiboscore	×		×	×	
Lid margin abnormalities	×		×	×	
Safety					
VAS	×	×	×		

#### 2.1.1 Ethics and dissemination

The protocol received endorsement from the Medical Ethical Committee of the Third Affiliated Hospital of Zhejiang Chinese Medical University, under the approval number ZSLL-KY-2023-049-01. Findings of the trial will be disseminated through peer-reviewed journals and scientific conferences.

#### 2.1.2 Informed consent

The study will be ensured adherence to the Declaration of Helsinki formulated by the World Medical Association and relevant clinical research regulations in China. Consent for participation, manifested through the signing of the informed consent form, must be secured before randomization. Participants retain the right to withdraw from the trial at any time, with the reasons for withdrawal meticulously recorded.

### 2.2 Participant recruitment

Recruitment began on December 12, 2024, and is expected to complete on December 20, 2025. Recruitment tactics will incorporate poster advertisements, community engagements, digital recruitment, and Ophthalmology outpatient services. Prospective participants will be briefed by their ophthalmologists regarding the study’s objectives, interventions, timeline, advantages, and potential risks at the designated hospitals. This will be followed by further verbal and written explanations from a local researcher. The trial is currently in the intervention phase with protocol version V3.0 and date December 2024.

#### 2.2.1 Inclusion criteria

Participants who meet the following inclusion criteria will be eligible for the study:

1. Confirmed diagnosis of DED in line with the 2021 Chinese Expert Consensus on Dry Eye (30): having at least one subjective symptom (e.g., dryness, foreign body sensation, burning sensation, fatigue, discomfort, fluctuating visual acuity) and SIT≤5 mm/5 min or non-invasive tear film breakup time (NIBUT) ≤ 5 s, or having at least one subjective symptom and 5 mm/5 min < SIT≤10 mm/5 min or 5 s < NIBUT≤10s, together with positive CFS;
2. Individuals aged 18 to 75 years, regardless of gender;
3. Absence of systemic medication intake within the previous two weeks;
4. Demonstrated capacity to cooperate during eye examinations and a commitment to participate in clinical observation trials;
5. Execution of the written informed consent form for participation in the clinical trial.

#### 2.2.2 Exclusion criteria

Participants who meet the following exclusion criteria will be excluded from the study:

1. Patients with hemorrhagic diseases;
2. Inability to adhere to the designated follow-up protocol;
3. Long-term users of sedatives, anti-depression, and anti-anxiety drugs;
4. Pregnancy or lactation;
5. Active ocular infection or inflammation unrelated to DED;
6. Ocular surgery or trauma within the past 6 months;
7. Severe meibomian gland dysfunction requiring procedural treatment;
8. Contact lens use within the past month;
9. Autoimmune diseases affecting the ocular surface.

### 2.3 Randomization and blinding

The random allocation sequence will be generated using the computer-based random number generation function in SPSS 25.0, individuals assigned odd numbers will receive acupuncture, while those with even numbers will receive medication. An independent statistician will generate a permuted-block sequence stratified by center. Group assignments will be placed in sequentially numbered, opaque, sealed envelopes. Enrolling clinicians will open envelopes after consent. Given acupuncture’s unique nature, only single-blinding is achievable in this study. Distinct roles of operator, assessor, and statistician are delineated to ensure unbiased efficacy assessment and data collection, aiming to minimize potential biases as effectively as possible. Outcome assessors and statisticians will remain blinded to group allocation.

### 2.4 Application selection

In this study, acupuncture needles are supplied by Huatuo, a brand produced by Suzhou Medical Supplies Factory, China. The manufacturer possesses a Manufacturer’s License Number: Su Drug Administration of Machinery Production 20,010,020 and a Registration Certificate Number: 20162200970. The needles come in two sizes: 0.18 × 25 mm and 0.25 × 40 mm. Ear press seeds are obtained from Shanghai TAICHENG Technology Development Co., Ltd. The sodium hyaluronate eye drops are from the Chinese brand Wanhan, holding a Chinese medicine approval No. H20203255. Comprehensive OS analyzer will utilize the MYAH ophthalmic optical biometry instrument from Topcon Healthcare Company, which is registered under Imported Medical Device Registration Number: 20232160188, and Product Serial Number: 172230032. The fluorescein sodium test strips are produced by Tianjin Jingming New Technological Development Co., Ltd. The manufacturer has a Manufacturer’s License Number: Jin Drug Administration of Machinery Production 20,100,040 and a Registration Certificate Number: 20222160497.

### 2.5 Interventions and comparison

#### 2.5.1 Intervention group

**Figure 2:**
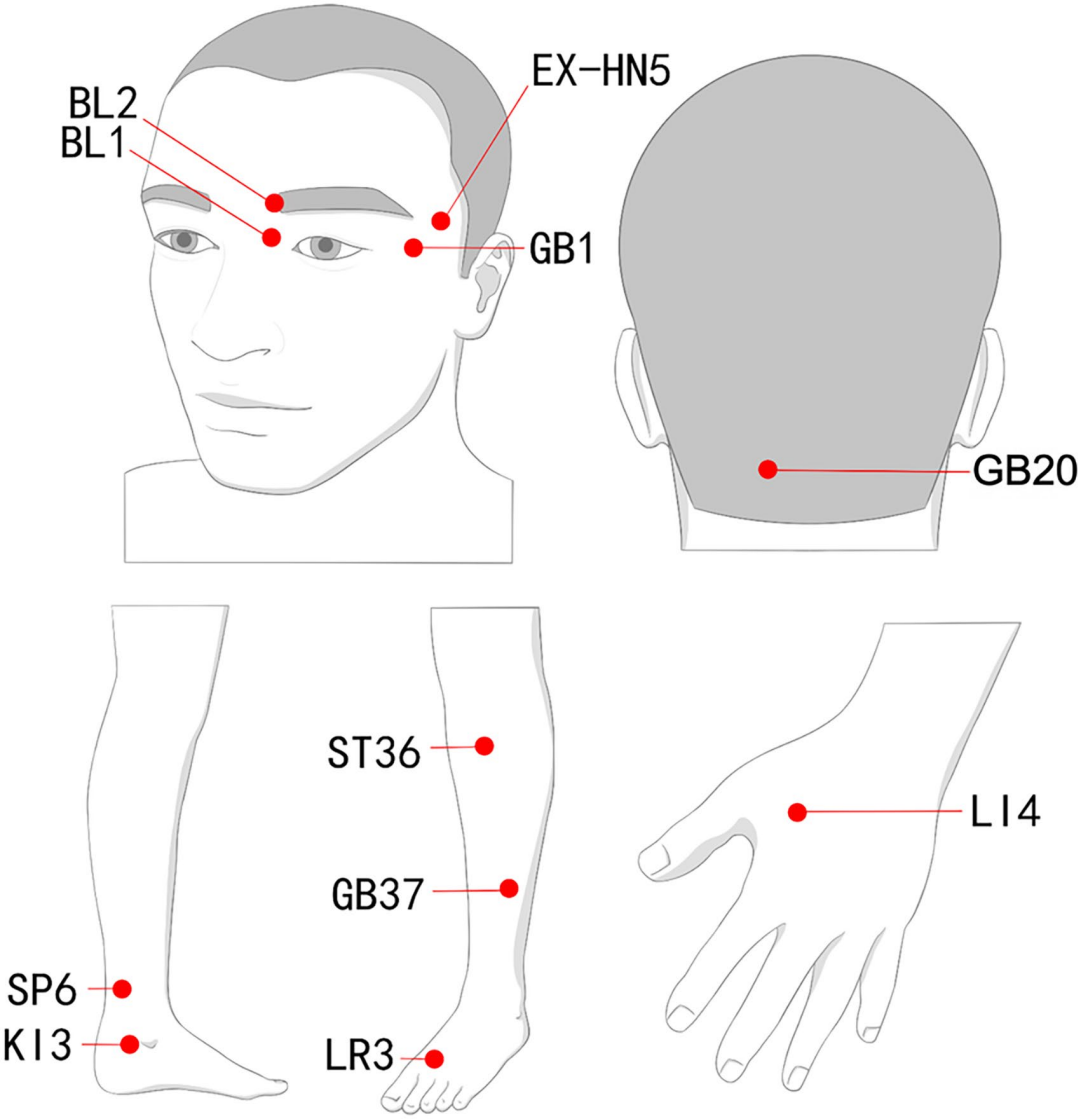
Location of acupoints.

Participants will receive the acupuncture combined with auricular acupressure. Acupuncture will be conducted bilaterally at specific acupoints based on published literature and textbooks about acupuncture for ophthalmologic diseases or dry eye syndrome (31, 32): Taiyang (EX-HN5), Jingming (BL1), TongZiliao (GB1), Cuanzhu (BL2), Fengchi (GB20), Taixi (KI3), SanYinjiao (SP6), Guangming (GB37), ZuSanli (ST36), Hegu (LI4), and Taichong (LR3). The techniques include: targeting EX-HN5 toward the outer canthus to a depth of 0.6 inch, inserting BL1 along the orbital rim until soreness is felt, penetrating GB1 straight to 1.0 inch and then redirecting it flat toward the ear tip after inducing soreness and numbness, and aligning BL2 in the direction of BL1 up to 0.6 inch. GB20 will be aimed toward the ipsilateral inner canthus, incorporating lifting, inserting, and slight rotating until a radiating sensation is felt in the forehead or eyes. Smaller needles (0.18 × 25 mm) are designated for EX-HN5, BL1, GB1, and BL2, whereas larger needles (0.25 × 40 mm) are reserved for the remaining points to achieve the “De Qi” sensation. “De Qi”is defined as a composite sensation including soreness, numbness, heaviness, or distension perceived by the participant and confirmed by the practitioner. The therapy will occur thrice weekly for 8 weeks, each session lasting 30 min, culminating in 24 sessions. The number of needles per session will range from 10 to 14, depending on bilateral point selection. Adherence will be monitored through treatment logs, and auricular seeds will be checked and replaced every 2 days if detached. All procedures will be executed by acupuncturists with at least 3 years of experience, following standardized training. Acupoint locations are based on the 2010 World Health Organization (WHO) guidelines (ISBN:9787117123327) (Figure 2; Table 2).

**Table 2:** Location of acupoints.

Acupoints	Location
Taiyang (EX-HN5)	In the region of the temples, in the depression about one fingerbreadth posterior to the midpoint between the lateral end of the eyebrow and the outer canthus.
Jingming (BL1)	In the depression superior to the inner canthus.
TongZiliao (GB1)	0.5 cun (Chinese inches) lateral to the outer canthus on the lateral side of the orbit.
Cuanzhu (BL2)	On the face, in the depression on the medial end of eyebrow, on the supraorbital notch.
Fengchi (GB20)	On the nape, below the occiput, in the depression between the upper portion of m. sternocleidomastoideus and m. trapezius.
Taixi (KI3)	Posterior to the medial malleolus, in the depression between the tip of the medial malleolus and tendo calcaneus.
Guangming (GB37)	5 cun (Chinese inches) above the tip of the lateral malleolus, on the anterior border of the fibula.
Hegu (LI4)	Located on the dorsum of the hand, between the 1st and 2nd metacarpal bones, in the middle of the 2nd metacarpal bone on the radial side.
Sanyinjiao (SP6)	Located on the inner side of the lower leg, 3 cun (Chinese inches) above the tip of the medial malleolus, just behind the posterior border of the tibia.
Zusanli (ST36)	3 cun (Chinese inches) below the lateral eye of the knee, at a point one horizontal finger’s width from the outer edge of the anterior tibial crest.
Taichong (LR3)	On the dorsum of the foot, in the depression proximal to the first metatarsal space.

**Figure 3:**
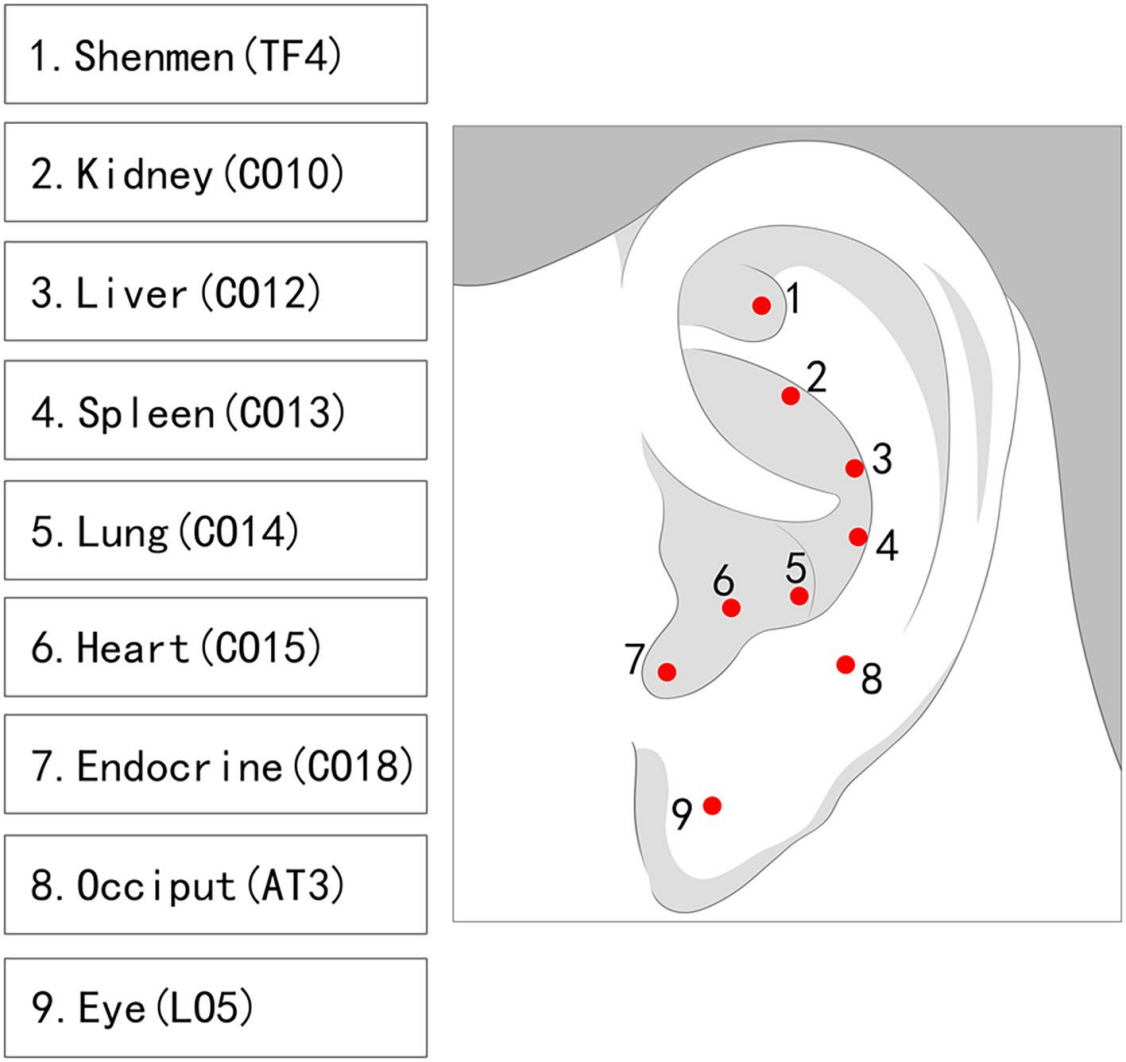
Location of auricular points.

Post-acupuncture, auricular acupressure will be applied and maintained for 2 days at points based on findings from previous studies (31, 33), including Eye (LO5), Heart (CO15), Liver (CO12), Spleen (CO13), Lung (CO14), Kidney (CO10), Occiput (AT3), Shenmen (TF4), and Endocrine (CO18). Participants will be instructed to press each auricular point 3–5 times per day, with each pressing lasting approximately 30–60 s. The intensity of stimulation will be standardized as a moderate, tolerable sensation, characterized by local soreness, distension, or warmth without causing pain. Participants will be instructed to avoid excessive force and to discontinue pressing if significant discomfort occurs. Auricular seeds will be replaced at the subsequent treatment session (Figure 3).

#### 2.5.2 Control group

Participants will receive 0.1% sodium hyaluronate eye drops (Wanhan), administered four times daily, one drop per eye, continuously for 8 weeks. Researchers will provide standardized instructions for eye drop application to ensure correct usage (33).

Both groups will abstain from glucocorticoids, nonsteroidal anti-inflammatory drugs, and related hormonal eye drops during the study. Use of additional lubricants is clarified and will be recorded. All concomitant medications will be meticulously recorded in the Case Report Form (CRF), noting the drug name, total daily dose, intended use, start and end dates, and usage status at the study’s conclusion. This trial adopts an active comparator rather than sham acupuncture, reflecting a pragmatic effectiveness design intended to evaluate the comparative benefit of acupuncture combined with auricular acupressure against commonly used standard therapy. This approach prioritizes clinical relevance and external validity, while acknowledging the potential influence of expectancy effects.

#### 2.5.3 Sample size

The sample size is based on the between-group difference in OSDI at week 8, and that longitudinal correlation is handled analytically via LMMs. According to the previous study (34), A 10-point reduction is adopted as the minimal clinically important difference (MCID). We assumed an SD = 18 for change in OSDI when powering the study based on previous experiment design (35). Thus, substituting *σ* = 18, *Δ* = 10, with two-sided *α* = 0.05, power = 80%, and a 20% attrition allowance, utilizing the formula: $n=\frac{2\sigma^{2}{\left(Z_{1-\alpha /2}+Z_{1-\beta }\right)}^{2}}{\Delta^{2}}.$ We calculated that 63 cases will be needed in each group, totaling 126. Each of the three sub-centers will recruit 21 participants for both the control and treatment groups.

### 2.6 Outcome measures

All outcome measures and their corresponding assessment timelines are specified in Table **1**, and the research protocol is depicted in Figure 3. Patients are required to abstain from using eye drops 4 hours before tests to prevent any immediate effects.

#### 2.6.1 Primary outcome

##### 2.6.1.1 OSDI

The primary outcome is the change in OSDI score from baseline to week 8. The OSDI, created by the International Dry Eye Workgroup, evaluates the subjective symptoms of DED using a 12-item questionnaire scored from 0 to 100, where higher scores denote more severe symptoms. The validated Chinese version of the OSDI (C-OSDI) will be used. A reduction of ≥10 points will be considered a clinically meaningful response (27).

#### 2.6.2 Secondary outcomes

The secondary ocular surface outcomes will be assessed at baseline and at weeks 8, 12, and analyzed primarily as changes from baseline using predefined continuous or ordinal scales. And the sleep quality and mood status will be evaluated at baseline, week 4, 8, 12, and week 16. Analyses will be based on changes in total scale scores from baseline.

##### 2.6.2.1 BUT

BUT quantifies the duration until the appearance of the first dry spot on the tear film following a complete blink, and is a critical measure of tear film stability (36). Fluorescein strips moistened with sterile saline will be applied in the inferior fornix. After three natural blinks, tear film break-up will be recorded under cobalt blue illumination. The average of three measurements will be used. Also, non-invasive TBUT will be reported to avoid fluorescein-induced artifacts.

##### 2.6.2.2 CIC

CIC is employed to gather cells from the OS for analyzing cytological alterations linked to DED. After administering topical anesthesia, a cellulose acetate membrane is used to collect cells from the superior temporal quadrant of the conjunctiva. These samples are stained with periodic acid-Schiff to evaluate the density and morphology of conjunctival goblet cells, identifying features like decreased goblet cell density, increased nuclear-cytoplasmic ratio, squamous metaplasia, and conjunctival epithelialization (37).

##### 2.6.2.3 CFS

CFS assesses the health of the corneal epithelium (38). We have specified the grading scales used (NEI 0–12 for CFS), inter-rater calibration procedures, and masking of assessors. The corneal surface is stained with fluorescein sodium, and then examined under cobalt blue light using a slit lamp. Each quadrant of the cornea (superior, inferior, nasal, and temporal) is scored from 0 to 3 based on staining severity, with total scores ranging from 0 to 12 for each eye.

##### 2.6.2.4 PSQI

The PSQI, a self-administered questionnaire, measures SQ over the preceding month (39). It includes seven components: SQ, sleep latency, sleep duration, habitual sleep efficiency, sleep disturbances, use of sleep medication, and daytime dysfunction. Higher composite scores indicate poorer SQ (40).

##### 2.6.2.5 SDS

The SDS consists of 20 questions rated on a 4-point scale, designed to assess the intensity of depressive symptoms. Higher scores suggest more severe symptoms.

##### 2.6.2.6 SAS

Similar to the SDS, the SAS measures anxiety levels and is scored to reflect the severity of anxiety symptoms. Higher scores indicate increased anxiety.

##### 2.6.2.7 TMH

TMH, measured using a comprehensive OS analyzer (MYAH), reflects the basal tear volume at the lower eyelid margin. A TMH of ≤0.2 mm serves as a diagnostic benchmark for dry eye (41). TMH will be measured non-invasively at the center of the lower eyelid margin under standardized ambient illumination. Three consecutive measurements will be obtained for each eye, and the mean value will be used for analysis. TMH values will be recorded in millimeters.

##### 2.6.2.8 MQS

MQS evaluates the nature of meibomian gland secretions, with scoring from 0 (clear, transparent liquid) to 3 (thick, toothpaste-like secretion). Each eyelid is assessed independently, and scores of 1 or higher are considered abnormal (42).

##### 2.6.2.9 Function of the meibomian gland

This evaluation encompasses five criteria: pachyblepharosis, irregularity of the eyelid margin, obstruction of the meibomian gland opening, congestion of the palpebral margin vessels, and anterior displacement of the skin-mucosal junction at the eyelid margin. Each item is scored as 1, for a total score range of 0 to 5 (43).

##### 2.6.2.10 MGS

MGS rates the expressibility and quality of meibum from 15 glands on the lower eyelid. Each of three sections on the eyelid—nasonasal, central, and temporal—is examined, assessing the ease of secretion and its characteristics (44). Each gland will be graded on a 0–3 scale (0 = clear secretion, 1 = cloudy, 2 = granular, 3 = inspissated or absent secretion), yielding a total score ranging from 0 to 15. A score of 3 or above is deemed abnormal (45).

##### 2.6.2.11 Meiboscore

To evaluate atrophy in the meibomian glands, keratography will be employed to obtain images of both upper and lower glands. Based on Arita (46), gland loss is classified using the meiboscore system as follows: grade 0 (no loss), grade 1 (less than one-third total gland area lost), grade 2 (one-third to two-thirds lost), and grade 3 (more than two-thirds lost). Average scores for both upper and lower eyelids will be computed, with scores ranging from 0 to 3.

TMH, MGS, meiboscore, and meibum quality will be assessed using a non-contact ocular surface analyzer (the MYAH ophthalmic optical biometry instrument from Topcon Healthcare Company) at baseline, week 8, and week 12 (47). All ocular surface images will be stored digitally and graded by two independent, trained ophthalmologists who are blinded to treatment allocation and visit sequence. In cases of disagreement, a third senior grader will adjudicate. Inter-rater reliability will be assessed prior to study initiation. The eye with the worse baseline OSDI-related ocular surface parameters (shorter TBUT or lower TMH) will be designated as the analysis eye. If both eyes meet identical criteria, the right eye will be selected. Only the analysis eye will be included in the primary analysis.

### 2.7 Statistical analysis

All analyses will follow a pre-specified statistical analysis plan (SAP) with two-sided tests at *α* = 0.05 and 95% CIs. The intention-to-treat (ITT) population is primary, with per-protocol (PP) analyses supportive and a safety set including all treated participants. Baseline comparability will be summarized with standardized mean differences (SMDs); if inferential testing is required, t-tests or Mann–Whitney U will be used for continuous variables and χ^2^ or Fisher’s exact for categorical variables. The primary endpoint (change in OSDI to Week 8) will be analyzed using a linear mixed-effects model (LMM) with fixed effects for group, time, and group×time, adjustment for baseline value, a random intercept for participant, and center modeled (random effect; fixed-effect sensitivity); the within-subject covariance will be chosen by AIC, and the primary contrast is the adjusted between-group difference at Week 8. Continuous secondary outcomes will use analogous LMMs; ordinal and binary outcomes will use cumulative-link mixed and mixed-effects logistic models, respectively; multiplicity will be handled by a prespecified hierarchy for key secondary endpoints and Benjamini–Hochberg FDR (q = 0.05) for the remainder. Missing data will be addressed by maximum likelihood within mixed models and by multiple imputation (*m* = 50) in sensitivity analyses. Adverse events will be summarized by group with risk estimates and 95% CIs. Prior to database lock, a blinded data review will be conducted to assess data completeness, distributional assumptions, and protocol adherence without unmasking treatment allocation. A detailed SAP will be finalized.

#### 2.7.1 Quality control

To ensure familiarity with the implementation plan and standard operating procedures (SOPs), a training session will be conducted one month before the trial begins, aiming to sustain the clinical research’s reliability. An independent safety monitor, who is not involved in participant recruitment, treatment delivery, outcome assessment, or data analysis, will oversee trial safety. The safety monitor will review accumulating safety data at predefined intervals and provide recommendations regarding trial continuation, modification, or termination. Given the minimal-risk nature of acupuncture and auricular acupressure, a formal Data and Safety Monitoring Board (DSMB) is not planned; however, independent safety oversight will be maintained. All data collected will be verified to confirm their reliability and originality. Specialized personnel will oversee trial data collection and analysis to reduce bias. Clinical data management will be entrusted to a professional data management firm, and the quality of clinical research will be stringently audited monthly.

#### 2.7.2 Safety monitoring

All acupuncture procedures will follow strict aseptic techniques, including skin disinfection, use of single-use sterile needles, and approved sharps disposal. Adverse events (AEs) and serious adverse events (SAEs) will be defined and graded according to established acupuncture safety guidelines, with attribution assessed by the investigator (48). Vasovagal reactions, hematoma, infection, and local pain will be specifically monitored, and all SAEs will be reported within 24 h. These events will be thoroughly recorded, detailing their onset, severity, duration, interventions made, and outcomes, and will be systematically entered into the Case Report Form (CRF). Additionally, a Visual Analogue Scale (VAS) will be administered following each treatment session to evaluate the safety and tolerability of the acupuncture treatments.

### 2.8 Data management and monitoring

All study data will be collected and managed using a secure electronic data capture (EDC) system designed for clinical research. The EDC system will maintain a complete audit trail, including timestamps, user identification, and reasons for data modification, to ensure data integrity and traceability. Investigators must complete case report forms consistent with the study protocol. Data management, led by Dr. Guo Qin, ensures the clinical trial data’s authenticity, integrity, and accuracy. Upon the study’s conclusion, investigators will forward all completed and signed case-report forms to the data management center for consistency checks, and queries will be issued to resolve any data discrepancies. All personal information of the participants, including names, phone numbers, and addresses, will be kept confidential to avoid any potential disclosure.

## 3 Discussion

DED is a prevalent ophthalmic condition known for its low clinical cure rates and frequent recurrences, leading to physical, psychological, and social dysfunction. Despite the variety of dry eye manifestations, the fundamental pathophysiological factors, including inflammation, neuromodulation, apoptosis, and hormonal imbalances, remain consistent across different cases (3). Current therapies vary in effectiveness and are often associated with side effects. Notably, OS damage in DED patients is frequently exacerbated by improper medication use (49), and while medications aimed at DED’s pathophysiology may offer gradual relief, they are susceptible to resistance (50). Evidence suggests that many patients do not achieve symptom alleviation with anti-inflammatory drugs, which can also introduce adverse effects (51). This has spurred growing interest in exploring complementary and alternative medicines for DED management.

Acupuncture, a key component of TCM, has demonstrated substantial benefits in treating various diseases, including DED (52, 53). It is known to improve nerve reflex sensitivity (54), reduce inflammatory responses (55), and elevate the levels of vasoactive peptides in lacrimal gland tissue. Additionally, it increases acetylcholine in tears, stimulates lacrimal gland cells to effectively secrete proteins, water, and electrolytes, enhances tear production (56), and prevents apoptosis in lacrimal gland epithelial cells (16). Research (57) has shown that acupuncture outperforms artificial tears in stabilizing tear film, reducing OS damage, and improving visual function in DED patients. The effectiveness of auricular acupressure in treating DED has also been validated, with mechanisms that include boosting tear protein levels, regulating hormones, reducing pain intensity, increasing neuropeptide levels, altering acetylcholine levels in the lacrimal gland, and enhancing OS microcirculation (58, 59). Our prior studies confirm that acupoint stimulation significantly reduces OS discomfort in DED patients (17, 18), and multiple RCTs have corroborated the sustained benefits of acupuncture on improving symptoms and clinical parameters of DED (60, 61). We hypothesize that acupuncture combined with auricular acupressure may lead to improvements in dry eye-related symptoms, as measured by changes in the Ocular Surface Disease Index, and selected ocular surface parameters. The primary analysis will estimate between-group differences in OSDI change from baseline to week 8 using linear mixed-effects models, and results will be reported with 95% confidence intervals.

Ameliorating the reduced QoL and mood disorders associated with DED is a key focus of this study. Severe symptoms can significantly impair daily life, deteriorating QoL as symptom severity increases (26). Sleep disturbances, anxiety, and depression may induce immune system dysfunctions and alter inflammatory factors and cytokines, potentially affecting the progression of dry eye syndrome (62). The efficacy of acupuncture and auricular acupressure in managing sleep disturbances and psychological mood disorders is well established (63–65). Furthermore, alleviating DED symptoms has been shown to improve SQ and mitigate anxiety and depression (29), with RCT results affirming that acupuncture effectively eases these conditions in DED patients (66). Given these findings, we posit that acupuncture paired with auricular acupressure could address these concerns in DED patients, likely yielding a synergistic effect where the combined benefits surpass those of the individual treatments.

While acupuncture has demonstrated efficacy in treating DED, there is still a notable deficiency in high-quality, multicenter clinical trials with extensive sample sizes and prolonged follow-up that specifically evaluate SQ, depression, and anxiety among DED patients. Furthermore, there is an inadequate comprehensive assessment of these psychological aspects in DED sufferers. This extensive multicenter clinical trial, with its diverse patient population, is poised to produce results that are both reliable and statistically significant. The study is expected to enhance tear secretion and tear film stability, reduce symptoms, improve SQ, mitigate anxiety and depression, and ultimately elevate the overall QoL for DED patients, while ensuring good safety and tolerability. This might bring new light on the existing treatment landscape of DED.

This trial has several limitations that must be acknowledged. Firstly, due to the unique nature of the acupuncture procedure and the setting of the intervention, it is not possible to blind the acupuncturists or the participants. Second, the prolonged course of treatment and follow-up period may lead to participant dropout. Third, the use of an active comparator rather than sham acupuncture may increase expectancy effects and limit the ability to isolate specific effects of acupuncture. Despite these limitations, this trial is expected to provide pragmatic evidence regarding the potential role of acupuncture combined with auricular acupressure in the management of dry eye disease within routine clinical settings and to inform the design of future confirmatory trials.

Acupuncture, rooted in TCM, is recognized for its minimally invasive nature and potential therapeutic benefits. It represents a viable alternative treatment that could foster better patient adherence and minimal side effects, particularly beneficial in long-term therapeutic scenarios. The outcomes of this study are anticipated to furnish new clinical insights that substantiate the efficacy of DED treatments and facilitate the incorporation of TCM into contemporary ophthalmological practices. Moreover, the expected results are intended to offer safer and more efficacious treatment alternatives for patients, thus improving their QoL.

## References

[1] Chinese Branch of the Asian Dry Eye Society, Ocular Surface and Tear Film Diseases Group of Ophthalmology Committee of Cross-Straits Medicine Exchange Association, Ocular Surface and Dry Eye Group of Chinese Ophthalmologist Association. Chinese expert consensus on dry eye: definition and classification. Chin J Ophthalmol. (2020) 56:418–22. doi: 10.3760/cma.j.cn112142-20200316-00190

[2] Akpek EK Amescua G Farid M Garcia-Ferrer FJ Lin A Rhee MK . American Academy of ophthalmology preferred practice pattern cornea and external disease panel. Dry eye syndrome preferred practice pattern®. Ophthalmology. (2019) 126:P286–334. doi: 10.1016/j.ophtha.2018.10.023, 30366798

[3] Jones L Craig JP Markoulli M Karpecki P Akpek EK Basu S . TFOS DEWS III. Am J Ophthalmol. (2025):39. doi: 10.1016/j.ajo.2025.05.039 41276204

[4] Huang QZ Cheng R Zhou J Xiang ZY. A meta-analysis of the prevalence of dry eye in Chinese adolescents under 18 years old. World Clin Drugs. (2022) 43:449–53. doi: 10.13683/j.wph.2022.04.015

[5] Craig JP Nelson JD Azar DT Belmonte C Bron AJ Chauhan SK . TFOS DEWS II report executive summary. Ocul Surf. (2017) 15:802–12. doi: 10.1016/j.jtos.2017.08.003, 28797892

[6] Yu J Asche CV Fairchild CJ. The economic burden of dry eye disease in the United States: a decision tree analysis. Cornea. (2011) 30:379–87. doi: 10.1097/ICO.0b013e3181f7f363, 21045640

[7] O'Neil EC Henderson M Massaro-Giordano M Bunya VY. Advances in dry eye disease treatment. Curr Opin Ophthalmol. (2019) 30:166–78. doi: 10.1097/ICU.0000000000000569, 30883442 PMC6986373

[8] He JJ Wang Y Zhao Y. Physical therapy of meibomian gland dysfunction and its progress. IES. (2019) 19:1146–9.

[9] Ferrari G Colucci A Barbariga M Ruggeri A Rama P. High frequency electrotherapy for the treatment of Meibomian gland dysfunction. Cornea. (2019) 38:1424–9. doi: 10.1097/ICO.0000000000002063, 31356415

[10] Cheng K Law A Guo MH Wieland LS Shen XY Lao LX. Acupuncture for acute hordeolum. Cochrane Database Syst Rev. (2017) 2:CD011075. doi: 10.1002/14651858.CD011075.pub2, 28181687 PMC5378315

[11] Law SK Wang L Li T. Acupuncture for glaucoma. Cochrane Database Syst Rev. (2020) 2:CD006030. doi: 10.1002/14651858.CD006030.pub4 32032457 PMC7006956

[12] Wang Y Gao YX Sun Q Bu Q Shi J Zhang YN . Acupuncture for adolescents with mild-to-moderate myopia: study protocol for a randomized controlled trial. Trials. (2014) 15:477. doi: 10.1186/1745-6215-15-477, 25476698 PMC4289244

[13] Liu M Liu ML Yu ML Lan L Yin HY Luo L . Acupuncture therapy for dry eye: a systematic review. Zhonghua Yan Ke Za Zhi. (2012) 22:242–6. doi: 10.13444/j.cnki.zgzyykzz.002994

[14] Wang ZL He HQ Hang D Shi CG. Effect of integral syndrome differentiation acupuncture on the tear film stability in the patient of xerophthalmia. Zhongguo Zhen Jiu. (2005) 25:460–3. doi: 10.13703/j.0255-2930.2005.07.005 16309130

[15] Xu Q Wei QB Ding N Shen HX Gao WP. Study on anti-inflammatory mechanism of acupuncture on dry eye based on p38 mitogen-activated protein kinase signaling pathway. J Tradit Chin Med. (2021) 36:5210–4.

[16] Zhang YM Gao WP. Acupuncture castrated rabbit dry eye model Schirmer and related proteins of the lacrimal gland epithelial cells Fas / FasL expression. J Liaoning Univ Tradit Chin Med. (2012) 14:248–50.

[17] Liu J Han DX Wang C Chen LF Wang CY Fang JQ. Clinical study on therapentic effect of dry eye syndrome treated with acupoint catgut implantation treatment. J Tradit Chin Med. (2020) 35:476–9.

[18] Liu J Lu TT Han DX Wang C Chen LF Wang CY . Dry eye syndrome of deficient lacrima productiontreated with the acupoint thread-embedding therapy: a randomized controlled trial. Zhongguo Zhen Jiu. (2019) 39:721–5. doi: 10.13703/j.0255-2930.2019.07.011, 31286734

[19] Zhang X He B Wang H Sun X. Auricular acupressure for treating early stage of knee osteoarthritis: a randomized, sham-controlled prospective study. QJM. (2022) 115:525–9. doi: 10.1093/qjmed/hcab230, 34463759

[20] Nogier R. History of auriculotherapy: additional information and new developments. Med Acupunct. (2021) 33:410–9. doi: 10.1089/acu.2021.0075, 34976274 PMC8716479

[21] Tan JY Molassiotis A Suen LKP Liu J Wang T Huang HR. Effects of auricular acupressure on chemotherapy-induced nausea and vomiting in breast cancer patients: a preliminary randomized controlled trial. BMC Complement Med Ther. (2022) 22:87. doi: 10.1186/s12906-022-03543-y, 35331208 PMC8953362

[22] Kong X Fang H Li X Zhang Y Guo Y. Effects of auricular acupressure on dysmenorrhea: a systematic review and meta-analysis of randomized controlled trials. Front Endocrinol. (2023) 13:1016222. doi: 10.3389/fendo.2022.1016222, 36686444 PMC9851274

[23] Mosavi Z Khazaie H Janatolmakan M Rezaeian S Khatony A. Effects of auricular acupressure on test anxiety in medical students: a randomized parallel-group trial. BMC Med Educ. (2023) 23:835. doi: 10.1186/s12909-023-04825-w, 37936159 PMC10629063

[24] Chen CH Chen HH Yeh ML Tsay SL. Effects of ear acupressure in improving visual health in children. Am J Chin Med. (2010) 38:431–9. doi: 10.1142/S0192415X10007956, 20503462

[25] Lee JS Hwang SH Shin BC Park YM. Electrical stimulation of auricular acupressure for dry eye: a randomized controlled-clinical trial. Chin J Integr Med. (2017) 23:822–8. doi: 10.1007/s11655-016-2449-6, 27080998

[26] Ayaki M Tsubota K Kawashima M Kishimoto T Mimura M Negishi K. Sleep disorders are a prevalent and serious comorbidity in dry eye. Invest Ophthalmol Vis Sci. (2018) 59:DES143–50. doi: 10.1167/iovs.17-23467, 30481819

[27] Zhang XM Yang LT Zhang Q Fan QX Zhang C You Y . Reliability of Chinese web-based ocular surface disease index questionnaire in dry eye patients: a randomized, crossover study. Int J Ophthalmol. (2021) 14:834–43. doi: 10.18240/ijo.2021.06.07, 34150537 PMC8165610

[28] Lu Y Zhu H Wang Q Tian C Lai H Hou L . Comparative effectiveness of multiple acupuncture therapies for primary insomnia: a systematic review and network meta-analysis of randomized trial. Sleep Med. (2022) 93:39–48. doi: 10.1016/j.sleep.2022.03.012, 35405419

[29] Bitar MS Olson DJ Li M Davis RM. The correlation between dry eyes, anxiety and depression: the Sicca, anxiety and depression study. Cornea. (2019) 38:684–9. doi: 10.1097/ico.0000000000001932, 30950896

[30] Chinese Branch of the Asian Dry Eye Society; Ocular Surface and Tear Film Diseases Group of Ophthalmology Committee of Cross-Straits Medicine Exchange Association; Ocular Surface and Dry Eye Group of Chinese Ophthalmologist Association. Expert consensus on dry eye in China: dry eye related to eye surgery (2021). Zhonghua Yan Ke Za Zhi. (2021) 57:564–72. doi: 10.3760/cma.j.cn112142-20210429-00196 34344116

[31] Qu MH Liu WL. (2018). Detailed explanation of the use of the latest national standard acupuncture points. Beijing: China Press of Traditional Chinese Medicine.

[32] Kim BH Kim MH Kang SH Nam HJ. Optimizing acupuncture treatment for dry eye syndrome: a systematic review. BMC Complement Altern Med. (2018) 18:145. doi: 10.1186/s12906-018-2202-0, 29724255 PMC5934900

[33] Q H M Z Z H. Auricular acupressure for dry eye disease: a systematic review and meta-analysis of randomized controlled trials. Med. (2023) 59:177. doi: 10.3390/medicina59010177 PMC9865136 36676806

[34] Miller KL. Minimal clinically important difference for the ocular surface disease index. Arch Ophthalmol. (2010) 128:94. doi: 10.1001/archophthalmol.2009.356 20065224

[35] Asbell PA Maguire MG Peskin E Bunya VY Kuklinski EJ. Dry eye assessment and management (DREAM©) study: study design and baseline characteristics. Contemp Clin Trials. (2018) 71:70–9. doi: 10.1016/j.cct.2018.06.002, 29883769 PMC7250048

[36] Ortiz-Toquero S Sanchez I Martin R. Effects of artificial tears on ocular surface symptoms and visual task performance in digital device users. Sci Rep. (2025) 16:653. doi: 10.1038/s41598-025-22510-4, 41453934 PMC12780262

[37] Nelson JD Havener VR Cameron JD. Cellulose acetate impressions of the ocular surface: dry eye states. Arch Ophthalmol. (1983) 101:1869–72. doi: 10.1001/archopht.1983.01040020871007, 6651590

[38] Pflugfelder SC. Advances in the diagnosis and management of keratoconjunctivitis sicca. Curr Opin Ophthalmol. (1998) 9:50–3. doi: 10.1097/00055735-199808000-00009 10387469

[39] Buysse DJ Reynolds CF Monk TH Berman SR Kupfer DJ. The Pittsburgh sleep quality index: a new instrument for psychiatric practice and research. Psychiatry Res. (1989) 28:193–213. doi: 10.1016/0165-1781(89)90047-4, 2748771

[40] Backhaus J Junghanns K Broocks A Riemann D Hohagen F. Test–retest reliability and validity of the Pittsburgh sleep quality index in primary insomnia. J Psychosom Res. (2002) 53:737–40. doi: 10.1016/s0022-3999(02)00330-6, 12217446

[41] Sang X Li Y Yang L Liu JH Wang XR Li CY . Lipid layer thickness and tear meniscus height measurements for the differential diagnosis of evaporative dry eye subtypes. Int J Ophthalmol. (2018) 11:1496–502. doi: 10.18240/ijo.2018.09.11, 30225224 PMC6133902

[42] Chinese Branch of the Asian Dry Eye Society, Ocular Surface and Tear Film Diseases Group of Ophthalmology Committee of Cross-Straits Medicine Exchange Association, Ocular Surface and Dry Eye Group of Chinese Ophthalmologist Association. Chinese expert consensus on meibomian gland dysfunction: diagnosis and management (2023). Chin J Ophthalmol. (2023) 59:880–7. doi: 10.3760/cma.j.cn112142-20230822-00054 37936356

[43] Nichols KK Foulks GN Bron AJ Glasgow BJ Dogru M Tsubota K . The international workshop on meibomian gland dysfunction: executive summary. Invest Ophthalmol Vis Sci. (2011) 52:1922–9. doi: 10.1167/iovs.10-6997a, 21450913 PMC3072157

[44] Arita R Minoura I Morishige N Shirakawa R Fukuoka S Asai K . Development of definitive and reliable grading scales for Meibomian gland dysfunction. Am J Ophthalmol. (2016) 169:125–37. doi: 10.1016/j.ajo.2016.06.025, 27345733

[45] Toyos R Desai NR Toyos M Dell SJ. Intense pulsed light improves signs and symptoms of dry eye disease due to meibomian gland dysfunction: a randomized controlled study. PLoS One. (2022) 17:e0270268. doi: 10.1371/journal.pone.0270268, 35737696 PMC9223330

[46] Arita R Itoh K Inoue K Amano S. Noncontact infrared meibography to document age-related changes of the meibomian glands in a normal population. Ophthalmology. (2008) 115:911–5. doi: 10.1016/j.ophtha.2007.06.031, 18452765

[47] Lal B Cantrell A Ostrin LA. Repeatability and agreement of the MYAH and Lenstar. Optom Vis Sci. (2024) 101:157–63. doi: 10.1097/OPX.0000000000002113, 38546757 PMC10987055

[48] Huang CC Kotha P Tu CH Huang MC Chen YH Lin JG. Acupuncture: a review of the safety and adverse events and the strategy of potential risk prevention. Am J Chin Med. (2024) 52:1555–87. doi: 10.1142/S0192415X24500617, 39460372

[49] Kam KW Di Zazzo A De Gregorio C Narang P Jhanji V Basu S. A review on drug-induced dry eye disease. Indian J Ophthalmol. (2023) 71:1263–9. doi: 10.4103/IJO.IJO_2782_22, 37026257 PMC10276716

[50] Fu H Wang J Zhang F Tang Y Zhou H Wang C. Effect of acupuncture versus artificial tears for dry eye disease: a protocol for systematic review and meta-analysis. Medicine. (2020) 99:e21301. doi: 10.1097/MD.0000000000021301, 32791714 PMC7387016

[51] White DE Zhao Y Jayapalan H Machiraju P Periyasamy R Ogundele A. Treatment satisfaction among patients using anti-inflammatory topical medications for dry eye disease. Clin Ophthalmol. (2020) 14:875–83. doi: 10.2147/OPTH.S233194, 32256045 PMC7089601

[52] Yang Y Shen Z Wu Z Luo L Liu J Liu B. Strategy programming for acupuncture development along One-Belt-one-road countries. Zhongguo Zhen Jiu. (2017) 37:343–8. doi: 10.13703/j.0255-2930.2017.04.001, 29231582

[53] Dhaliwal DK Zhou S Samudre SS Lo NJ Rhee MK. Acupuncture and dry eye: current perspectives. A double-blinded randomized controlled trial and review of the literature. Clin Ophthalmol. (2019) 13:731–40. doi: 10.2147/OPTH.S175321, 31114151 PMC6497118

[54] Zhang YB Gao WP. Effect of acupuncture on microscopic morphology of lacrimal fluid of dry eye rabbit with hydropenia. J Nanjing TCM. (2010) 26:47–9. doi: 10.14148/j.issn.1672-0482.2010.01.011

[55] Lin ZS Yu DS Zhao JL Shi HY Zhang ZQ Zhao L . Effect of acupuncture on dry eye and tear inflammatory factors. Zhongguo Zhen Jiu. (2022) 42:1379–83. doi: 10.13703/j.0255-2930.20220318-0001, 36484191

[56] Zhang LL Zhang CH Ma XP Yang L Hong Y Wu LX . Current status and prospect of acupuncture and moxibustion in the treatment of dry eye syndrome. JCAM. (2014) 30:62–6.

[57] Zhang X Zhang B Peng S Zhang G Ma J Zhu W. Effectiveness of acupuncture at acupoint BL1 (Jingming) in comparison with artificial tears for moderate to severe dry eye disease: a randomized controlled trial. Trials. (2022) 23:605. doi: 10.1186/s13063-022-06486-4, 35897025 PMC9327344

[58] Gong L Sun X Chapin WJ. Clinical curative effect of acupuncture therapy on xerophthalmia. Am J Chin Med. (2010) 38:651–9. 20626051 10.1142/S0192415X10008123

[59] Pesavento F Lovato A Cappello S Postiglione M. Acupuncture in the treatment of dry eye syndrome with anxiety symptoms. A case report. Eur J Transl Myol. (2022) 32:10482. doi: 10.4081/ejtm.2022.10482, 35727219 PMC9295179

[60] Prinz J Maffulli N Fuest M Walter P Hildebrand F Migliorini F. Acupuncture for the management of dry eye disease. Front Med. (2022) 16:975–83. doi: 10.1007/s11684-022-0923-4, 36152126

[61] Fu Y Liu C Xu G. Mechanisms of acupuncture in the treatment of dry eye disease: narrative review of experimental studies. Int Ophthalmol. (2025) 45:464. doi: 10.1007/s10792-025-03833-7, 41196433

[62] Zhou Y Murrough J Yu Y Roy N Sayegh R Asbell P . Association between depression and severity of dry eye symptoms, signs, and inflammatory markers in the DREAM study. JAMA Ophthalmol. (2022) 140:392–9. doi: 10.1001/jamaophthalmol.2022.0140, 35266971 PMC8914873

[63] de Oliveira Rodrigues DM Menezes PR Machado Ribeiro Silotto AE Heps A Pereira Sanches NM Schveitzer MC . Efficacy and safety of auricular acupuncture for depression: a randomized clinical trial. JAMA Netw Open. (2023) 6:e2345138. doi: 10.1001/jamanetworkopen.2023.45138, 38032640 PMC10690462

[64] Smith CA Armour M Lee MS Wang LQ Hay PJ. Acupuncture for depression. Cochrane Database Syst Rev. (2018) 3:CD004046. doi: 10.1002/14651858.CD004046.pub4, 29502347 PMC6494180

[65] Kim SA Lee SH Kim JH van den Noort M Bosch P Won T . Efficacy of acupuncture for insomnia: a systematic review and meta-analysis. Am J Chin Med. (2021) 49:1135–50. doi: 10.1142/S0192415X21500543, 34049475

[66] Duan H Zhou Y Ma B Liu R Yang T Chu H . Effect of acupuncture treatment on the ocular pain, mental state and ocular surface characteristics of patients with dry eye disease: a non-randomized pilot study. Clin Ophthalmol. (2024) 18:2751–64. doi: 10.2147/OPTH.S476573, 39376907 PMC11457765

